# Neuropsychiatric Symptoms in Patients with the Main Etiological Types of Mild Neurocognitive Disorders: A Hospital-Based Case–Control Study

**DOI:** 10.3389/fpsyt.2017.00075

**Published:** 2017-05-04

**Authors:** Oleg A. Levada, Nataliya V. Cherednichenko, Alexandra S. Troyan

**Affiliations:** ^1^State Institution “Zaporizhzhia Medical Academy of Postgraduate Education Ministry of Health of Ukraine”, Zaporizhzhia, Ukraine

**Keywords:** mild neurocognitive disorder due to Alzheimer’s disease, subcortical vascular mild neurocognitive disorder, neuropsychiatric symptoms, irritability, anxiety, depression

## Abstract

**Background:**

The diagnostic construct of mild neurocognitive disorders (MNCDs) is substantially congruent with previously proposed criteria for mild cognitive impairment (MCI). MNCD/MCI is associated with neuropsychiatric symptoms (NPS). Previous studies have examined the prevalence of NPS in amnestic and non-amnestic MCI subtypes; however, no studies exist for etiological types of MNCD. We aimed to estimate the prevalence of NPS in patients with MNCD due to Alzheimer’s disease (MNCD-AD) and subcortical vascular MNCD (ScVMNCD) and to determine whether NPS would expand these MNCD phenotypes.

**Methods:**

The sample comprised 70 patients with MNCD-AD, 70 patients with ScVMNCD, and 55 cognitively normal elderly persons (CNEP). The diagnosis of MNCD-AD was made according to DSM-5 criteria for possible MNCD-AD. ScVMNCD patients fulfilled the DSM-5 criteria of the probable vascular MNCD and the diagnostic criteria for subcortical vascular MCI according to Frisoni et al. (1). The prevalence of NPS was based on the neuropsychiatric inventory. The statistical analyses included parametric and non-parametric tests, multivariate regression, and Spearman’s correlation coefficient.

**Results:**

About 69.1% of CNEP, 97.1% of MNCD-AD, and 100% of ScVMNCD patients had one or more NPS. The prevalence of NPS in both MNCD groups was significantly higher than that in CNEP. The most prevalent NPS that had significant differential diagnostic value in separating MNCD-AD from ScVMNCD, as well as MNCD from CNEP, were anxiety (81.43%) and irritability (67.14%) in MNCD-AD and depression (81.43%) in ScVMNCD. In both MNCD groups, we observed significant (*p* < 0.05) correlations between all distinguishing NPS and the differential cognitive disturbances: the amnestic syndrome in MNCD-AD and executive dysfunction in ScVMNCD.

**Conclusion:**

NPS occur in the majority of persons with MNCD-AD and ScVMNCD. Anxiety and irritability are the most prevalent NPS in MNCD-AD, as well as depression in ScVMNCD. The amnestic–anxious–irritable syndrome can be the main phenotype in MNCD-AD, on the other hand, the dysexecutive–depressive syndrome can be considered as the most prevalent clinical manifestation in ScVMNCD. Obtained data may be used for clinical differentiation of MNCD-AD and ScVMNCD patients.

## Introduction

The diagnostic construct of mild neurocognitive disorders (MNCDs) is substantially congruent with the previously proposed nosological entity for mild cognitive impairment (MCI) (2). It was shown that overlap between MNCD and MCI diagnosis is 98.6% (3). This clinical concept has been distinguished as an intermediate state between normal aging and dementia (4). Subjects with MCI constitute a high-risk group because they develop dementia at a rate of 10–15% per year as compared to 1–2% per year in the general population (5). A rich set of data regarding the occurrence, risk factors, and progression of MCI has been generated. Research indicates that features apart from cognition provide additional information about MCI phenotypes (6). Over the last decade, neuropsychiatric symptoms (NPS) in MCI have been described. Thus, the global prevalence of NPS in MCI ranges from 35 to 85% (7). Hospital-based samples have reported a higher global prevalence of NPS than population-based studies (6–8). According to a systematic review, the most common NPS are depression, anxiety, and irritability (7). The presence of NPS is strongly associated with a higher risk of cognitive and functional deterioration in MCI patients (9, 10). Prospective studies have shown that NPS, particularly depression, might represent risk factors for the conversion of MCI to Alzheimer’s disease (AD) (7) and vascular dementia (11).

Most of the previous investigations have examined the presence of NPS in clinical-based MCI subtypes—single-domain amnestic, multi-domain amnestic, single-domain non-amnestic, and multi-domain non-amnestic (8, 12). Nevertheless, analysis of NPS in the main etiological MCI types might have theoretical as well as practical value. Etiological DSM-5 criteria of MNCD open new perspectives for this purpose. According to epidemiological data, the main etiological type of MNCD/MCI is MNCD due to Alzheimer’s disease (MNCD-AD) and the second is subcortical vascular one [subcortical vascular MNCD (ScVMNCD)] (13). In this study, we aimed to estimate the prevalence of NPS in patients with MNCD-AD and ScVMNCD. We hypothesized that the additional consideration of NPS would expand these MNCD phenotypes.

## Materials and Methods

### Study Design

We conducted a case–control study comparing 70 patients with MNCD-AD to 70 patients with ScVMNCD and to 55 cognitively normal elderly persons (CNEP). All participants were outpatients of the neurological department of the Central Hospital of Kommunarsky District, Zaporizhzhia, Ukraine. All subjects were medically stable and did not have significant confounding neurological conditions, recent substance or alcohol abuse, or current primary psychiatric diagnoses. Patients with initial memory and other cognitive complaints were screened for further MNCD diagnosis confirming. The diagnosis of MNCD-AD was made according to DSM-5 criteria for possible MNCD-AD (2). Patients with ScVMNCD fulfilled the DSM-5 criteria of the probable neuroimaging-supported vascular MNCD and the diagnostic criteria for subcortical vascular MCI according to Frisoni et al. (1). Subjects with MNCD had a Clinical Dementia Rating (CDR) global score of 0.5 (14) and a Mini-Mental State Examination (MMSE) (15) score of 24–27 (inclusive) according to Hamrick et al. (16). MMSE has been shown to be superior to Montreal Cognitive Assessment for the estimation of cognitive decline in MNCD patients aged 75+ (17). Outpatients with a history of peripheral nervous system diseases were included into CNEP group. They had no cognitive complaints, a CDR global score of 0, and an MMSE score of 28–30. All of the participants were subjected to neuropsychological, neuropsychiatric, neurological, and functional evaluations. Moreover, MNCD patients underwent brain MRI to fulfill the criteria of MNCD-AD and ScVMNCD.

This study was approved by the local ethics committee and performed in accordance with the ethical standards laid down in the 1964 Declaration of Helsinki and its later amendments. All participants gave written informed consent prior to participation in the study.

### Neuropsychological Assessment

The neuropsychological investigation included CDR (14), MMSE (15), Luria’s tests (18), ten-minute intermediate memory evaluation (TIME) test (19), the clock-drawing test (20), and the verbal fluency test (21). All the assessments were done for the diagnosis of MNCD subtype according to DSM-5 criteria. Meanwhile, it was interesting to inner relationships between neuropsychological features of studied MNCDs and distinguishing NPS for assessing possible clinical phenotypes. Therefore, we also examined them as the variables of interest. The severity of impairments was evaluated on a scale of 0–3, where 0 meant “not impaired” and 3 meant “the most impaired” (for Luria’s tests) or according to author’s recommendations to the scales.

Ten-minute intermediate memory evaluation test consisted of three parts: a list-learning task, a semantic memory task, and a visual memory task. In the first one, a patient is told to remember five unrelated words, which are presented three times. Their immediate spontaneous recall across the three trials is recorded. Afterward, a semantic message including five elements is read and immediate recall requested. The message is repeated twice. Subsequently, a subject is asked to remember a drawing with five elements. This is succeeded with a spontaneous free recall, cued recall, and recognition of the five words and the semantic message. Further, the spontaneous recall of the figure is requested, followed by the recognition of the figure from among five designs.

### Neuropsychiatric Assessment

The neuropsychiatric inventory (NPI) (22) was administered to a spouse or another knowledgeable informant who could report the patient’s NPS. NPI collects information on symptoms during the past month in 12 neuropsychiatric domains, i.e., agitation, delusion, hallucination, depression, anxiety, euphoria, apathy, disinhibition, irritability, aberrant motor behavior, sleep, and eating/appetite. There is a “yes” or “no” screening question for each domain. If the respondent answers affirmatively, then additional information is obtained on the frequency (4-point scale), severity (3-point scale), and caregiver distress (6-point scale) associated with the behavior. Symptoms were defined as clinically significant if the product of the frequency and severity score of the symptom was 4 or higher according to Schneider et al. recommendations (23).

Neurological examination was conducted according to a standard protocol with a detailed assessment of gait—Tinetti performance-oriented mobility assessment (POMA) (24).

### Functional Assessment

Activities of daily living were assessed with the help of the Bristol Activities of Daily Living Scale (BADL) (25).

### Statistical Analysis

Statistical analysis was conducted using “STATISTICA 6.0” for Windows (StatSoft Inc., USA) v.6.1 and SPSS Statistics (IBM) v.20.0. The results were given as percentages, medians, and interquartile ranges or means and SDs, depending on the data and distribution. The statistical significance of between-group comparisons was determined using parametric and non-parametric criteria when appropriate (chi-squared test, Kruskal–Wallis test with subsequent multiple comparisons, ANOVA, *post hoc* Scheffe test). Additionally, we assessed whether NPS may predict group membership using multivariate logistic regression analysis when adjusted for confounders (age, sex, and education). We quantified the magnitude of the association between a specific NPS and MNCD membership by computing the odds ratio (OR) and the corresponding 95% confidence intervals (95% CI). Further, the relationships between assessed distinguishing NPS and prominent clinical parameters of MNCD patients were investigated using Spearman’s correlation coefficient (*r*_s_). Significance was set at *p* < 0.05.

## Results

Table 1 summarizes the main demographic, cognitive, neurological, and functional data of the comparison groups. Surveyed cohorts did not differ by age, gender, and level of education. All participants pertained to the age group of 65–91. All groups had a larger proportion of women than men, but differences in proportions between the groups were not significant. The mean education level of the CNEP and MNCD subjects was about 12 years. The severity of cognitive impairment (total MMSE score) corresponded to the previously obtained rates for those with MCI/MNCD. The MMSE total score was significantly lower in both MNCD-AD and ScVMNCD groups in comparison with control. Meanwhile, we found no significant difference in cognitive impairment between MNCD-AD and ScVMNCD groups.

**Table 1:** **Main demographic, cognitive, neurological, and functional characteristics of the comparison groups**.

Variables	Comparison groups			*p*-Value		
	CNEP (*n* = 55)	MNCD-AD (*n* = 70)	ScVMNCD (*n* = 70)	CNEP vs MNCD-AD	CNEP vs ScVMNCD	MNCD-AD vs ScVMNCD
Age (years)	74.36 ± 5.42	74.03 ± 5.95	74.09 ± 5.77	0.95^a^	0.97^a^	0.99^a^
Gender, male/female	19/36	25/45	32/38	0.89^b^	0.21^b^	0.23^b^
Education (years)	12.72 ± 2.32	12.18 ± 3.10	12.35 ± 2.93	0.58^a^	0.78^a^	0.94^a^
**Neuropsychological assessment**						
Mini-Mental State Examination, score	29 (28–30)	26 (25–26)	25 (25–26)	<0.00001	<0.00001	0.09
Delayed recall, ten-minute intermediate memory evaluation (TIME) test, number of words (0–5)	5 (4–5)	3 (2–3)	2 (2–3)	<0.00001	<0.00001	0.05
Delayed cued recall + recognition, TIME test, number of words (0–10)	9 (8–10)	5 (5–7)	8 (7–9)	<0.00001	<0.00001	<0.00001
Clock drawing test, part I, score (0–10)	10 (9–10)	9 (9–10)	8 (7–8)	0.09	<0.00001	<0.00001
Verbal fluency, number of words during 3 min	23 (22–26)	23 (16–24)	15 (13–17)	0.06	<0.00001	<0.00001
Kinetic apraxia, Luria’s tests, score (0–3)	0 (0–0)	0 (0–0)	2 (2–3)	1.0	<0.00001	<0.00001
Visuospatial apraxia, Luria’s tests, score (0–3)	0 (0–0)	1 (0–1)	0 (0–0)	<0.00001	0.75	0.0003
**Neurological assessment**						
The severity of pseudobulbar syndrome, score (0–3)	0 (0–0)	0 (0–0)	1 (1–1)	1.0	<0.00001	<0.00001
Performance-oriented mobility assessment, score (0–28)	28 (27–28)	27 (26–28)	19 (16–21)	0.06	<0.00001	<0.00001
**Functional assessment**						
Bristol Activities of Daily Living Scale, score (0–60)	0 (0–0)	1 (0–1)	2 (1–2)	<0.00001	<0.00001	0.00002

*Data are presented as mean ± SD/median (lower–upper quartile); CNEP, cognitively normal elderly persons; mild neurocognitive disorder (MNCD)-AD, patients with mild neurocognitive disorder due to Alzheimer’s disease (AD); subcortical vascular MNCD (ScVMNCD), patients with mild subcortical vascular neurocognitive disorder*.

^a^ANOVA, Post hoc Scheffe test

*^b^Chi-square test; non-parametric ANOVA test for multiple comparisons if not otherwise specified*.

The analysis revealed some significant clinical features that reliably distinguished patients with MNCD-AD and ScVMNCD. The most prominent feature of MNCD-AD patients was an amnestic syndrome, which was substantially more severe in comparison with CNEP. The severity of impairment of spontaneous delayed recall of five nouns (TIME-test) was comparable in MNCD-AD and ScVMNCD groups. However, patients with MNCD-AD did not significantly improve the results of recall during cued recall and recognition. Subjects with MNCD-AD also had mild visuospatial apraxia when performing Luria’s tests. This symptom differentiated MNCD-AD patients from controls and those with ScVMNCD, who did not have any visuospatial disturbances. We also found no significant neurological disturbances in MNCD-AD group. Similarly, patients with ScVMNCD showed distinct spontaneous delayed recall impairment, but they had significantly higher rates of cued verbal recall and recognition than those with MNCD-AD. In ScVMNCD group, we revealed pronounced executive and neurological disturbances in contrast to the CNEP and MNCD-AD subjects. The executive dysfunction manifested in performing the clock-drawing test (part I), the verbal fluency test, and Luria’s tests for kinetic apraxia. Moreover, there were mild-to-moderate pseudobulbar signs and frontal lobe gait disturbances (decreased POMA score) in ScVMNCD patients. On average, all MNCD patients had minimal disruption in everyday activities by BADL scale; however, it was significantly different from controls. Furthermore, ScVMNCD persons had more severe functional impairment compared to those with MNCD-AD.

Table 2 displays the frequency of NPS in CNEP, MNCD-AD, and ScVMNCD groups. About 69% of CNEP, 97% of MNCD-AD patients, and all (100%) participants with ScVMNCD had one or more NPS. Although the prevalence of NPS in both MNCD groups was significantly higher than in cognitively normal persons, there was no difference between MNCD-AD and ScVMNCD groups. The most prevalent NPS in patients with MNCD-AD that distinguished them from cognitively normal controls and ScVMNCD group were anxiety (81.43%), irritability (67.14%), and sleep disturbances (67.14%). The frequency of depression (28.57%) in MNCD-AD was significantly lower than in those with ScVMNCD and did not discriminate MNCD-AD patients from CNEP. The most distinguishing NPS in ScVMNCD group were depression (81.43%) and apathy (47.14%). They were present reliably more often than in CNEP and MNCD-AD group. Sleep disturbances in ScVMNCD were relatively frequent (31.43%); nonetheless, their frequency was lower than in MNCD-AD and it did not discriminate ScVMNCD persons from CNEP. Rare but distinguishing NPS in ScVMNCD was disinhibition (12.87%). Euphoria, aberrant motor behavior, and eating/appetite disturbances were rare events in patients with MNCD and absent in CNEP. Agitation, delusion, and hallucinations were not present in comparison groups.

**Table 2:** **Frequency of neuropsychiatric symptoms (NPS) in CNEP, and patients with the main types of mild neurocognitive disorder (MNCD)**.

Neuropsychiatric inventory (NPI) items	Comparison groups			*p*-Value		
	CNEP (*n* = 55) *n* (%)	MNCD-AD (*n* = 70) *n* (%)	ScVMNCD (*n* = 70) *n* (%)	CNEP vs MNCD-AD	CNEP vs ScVMNCD	MNCD-AD vs ScVMNCD
NPI, total score (0–144)	6 (3–9)	17 (12–21)	19 (14–23)	<0.00001^a^	<0.00001^a^	0.97^a^
Total prevalence of NPS	38 (69.09)	68 (97.1)	70 (100)	<0.00001	<0.00001	0.15
Agitation	0 (0)	0 (0)	0 (0)	–	–	–
Delusion	0 (0)	0 (0)	0 (0)	–	–	–
Hallucinations	0 (0)	0 (0)	0 (0)	–	–	–
Depression	8 (14.55)	20 (28.57)	57 (81.43)	0.062	<0.00001	<0.0001
Anxiety	27 (49.10)	57 (81.43)	21 (30.0)	<0.0001	0.03	<0.0001
Euphoria	0 (0)	0 (0)	1 (1.43)	–	0.37	0.32
Apathy	0 (0)	0 (0)	33 (47.14)	–	<0.00001	<0.00001
Disinhibition	0 (0)	0 (0)	9 (12.87)	–	0.006	0.002
Irritability	8 (14.55)	47 (67.14)	22 (31.43)	<0.0001	0.03	<0.0001
Aberrant motor behavior	0 (0)	0 (0)	4 (5.71)	–	0.07	0.04
Sleep	12 (21.82)	47 (67.14)	22 (31.43)	<0.0001	0.30	<0.0001
Eating/appetite	0 (0)	3 (4.29)	5 (7.14)	0.12	0.04	0.47

*Data are presented as median (lower–upper quartile)/number of patients (percentage)*.

*CNEP, cognitively normal elderly persons; MNCD-AD, patients with mild neurocognitive disorder due to Alzheimer’s disease; ScVMNCD, patients with mild subcortical vascular neurocognitive disorder*.

*^a^Non-parametric ANOVA test for multiple comparisons; chi-square test if not otherwise specified*.

Afterward, we calculated OR for each NPS of which significant difference was observed by chi-square test using logistic regression analysis comparing MNCD patients with CNEP and ScVMNCD with MNCD-AD group (Table 3). The most distinguishing features to predict MNCD membership were depression, irritability, and sleep in comparison with CNEP. Depression conveyed significant risk for ScVMNCD membership when compared with MNCD-AD, meanwhile anxiety and irritability conveyed significant risk for MNCD-AD comparing with ScVMNCD. Sleep disturbance was a frequent but non-specific symptom between MNCD-AD and ScVMNCD groups.

**Table 3:** **Odds ratio (OR) of the neuropsychiatric symptoms (NPS) of mild neurocognitive disorder (MNCD) groups**.

NPS	MNCD vs cognitively normal elderly persons			Subcortical vascular MNCD vs MNCD due to Alzheimer’s disease (MNCD-AD)		
	OR	95% CI	*p*	OR	95% CI	*p*
Depression	3.732	4.40–44.84	0.006	13.876	3.961–48.616	<0.0001
Apathy	>1,000	0–∞	0.998	>1,000	0–∞	0.998
Anxiety	1.81	0.511–2.730	0.697	0.205	0.064–0.659	0.008
Irritability	4.471	1.794–11.142	0.001	0.169	0.049–0.575	0.004
Sleep	3.236	1.354–7.730	0.008	0.925	0.304–2.818	0.891

*OR and 95% CI adjusted for age (continuous variable), sex, and education (continuous variable)*.

Further, we assessed possible links between relevant NPS in MNCD groups and associations between distinguishing NPS and other clinical features of MNCD. We hypothesized that the presence of such relationships might indicate common mechanisms of pathogenesis for NPS, cognitive and neurological syndromes of MNCD. We used correlation analyses for this purpose. For MNCD-AD group, the relevant NPS variables were anxiety and irritability, as well as a delayed recall score and visuospatial apraxia severity as the distinguishing cognitive signs. For ScVMNCD group, the relevant NPS, cognitive and neurological features were depression, a decrease in delayed recall performance, a clock-drawing test (part I) score, a verbal fluency test score, kinetic apraxia severity, and a POMA score (severity of gait disturbances). For both MNCD groups, we assessed correlations between total NPI score and each relevant NPS, total MMSE score, and total BADL score. Table 4 summarizes the results.

**Table 4:** **Spearman’s correlations between distinguishing neuropsychiatric symptoms and relevant cognitive, neurological, functional variables in mild neurocognitive disorder due to Alzheimer’s disease (MNCD-AD)/subcortical vascular MNCD (ScVMNCD) group**.

Clinical variables	MNCD-AD group			ScVMNCD group	
	Anxiety, neuropsychiatric inventory (NPI) score	Irritability, NPI score	NPI total score	Depression, NPI score	NPI total score
NPI total score	0.81*	0.71*	–	0.81*	–
Anxiety, NPI score	–	0.51*	0.81*	–	–
Irritability, NPI score	0.51*	–	0.71*	–	–
Depression, NPI score	–	–	–	–	0.81*
Mini-Mental State Examination, total score	−0.53*	−0.52*	−0.68*	−0.71*	−0.73*
Delayed recall, ten-minute intermediate memory evaluation (TIME) test	−0.36*	−0.33*	−0.52*	–	–
Clock drawing test, part I, score	–	–	–	−0.69*	−0.65*
Verbal fluency, number of words	–	–	–	−0.56*	−0.52*
Kinetic apraxia, Luria’s tests, score	–	–	–	0.75*	0.72*
Visuospatial apraxia, Luria’s tests, score	0.23*	0.24*	0.30*	–	–
Performance-oriented mobility assessment, total score	–	–	–	−0.66*	−0.64*
Bristol Activities of Daily Living Scale, score	0.33*	0.30*	0.44*	0.66*	0.67*

*p < 0.0.5.

In both MNCD groups, we observed significant (*p* < 0.05) positive moderate correlations between all distinguishing NPS, and between NPS and total NPI score. Significant associations were also found between all meaningful NPS and the differential clinical features in MNCD-AD and ScVMNCD patients. In MNCD-AD group, those correlations were predominantly moderate. In this group, anxiety and irritability had negative correlations with MMSE total score and memory disturbances (spontaneous delayed recall). In patients with ScVMNCD, there were strong negative correlations between depression and MMSE score and the clock-drawing test (part I) score. Depression positively correlated with kinetic apraxia severity (strong correlation) and negatively correlated with the verbal fluency test score (moderate correlation) as well as with frontal type gait disturbances (strong correlation). All significant NPS and total NPI score in both groups of MNCD positively correlated with deteriorations in daily living activities (BADL score). Those correlations were weak in MNCD-AD group and strong in patients with ScVMNCD. Moreover, we found statistically significant correlations between total NPI score and most of the cognitive and neurological disturbances in MNCD patients.

## Discussion

In this study, for the first time to our knowledge, we evaluated the prevalence of NPS in elderly patients with MNCD according to their etiological type. Subjects with MNCD-AD and ScVMNCD as well as cognitively normal persons were sampled from admitted to the neurological department of the Zaporizhzhia city hospital in Ukraine. Most of CNEP (69%), 97% of MNCD-AD, and 100% participants with ScVMNCD had one or more NPS. The prevalence of NPS in both MNCD groups was significantly higher than in cognitively normal persons. Our data confirm a high prevalence of NPS in MNCD reported by other population-based and hospital-based studies. In a systematic review by Monastero et al. (7), the global prevalence of NPS in MCI ranged from 35 to 85%. It was observed that hospital-based sample studies reported a higher global prevalence of NPS (26, 27) than population-based studies (6–8). The available hospital-based studies (26, 27) were conducted on heterogeneous samples of patients; therefore, we could not directly compare our data of NPS prevalence with their results. Moreover, clinical samples are often subject to referral bias; hence, it is difficult to compare our results with population-based samples (6, 8, 28–30).

In our study, we found the most distinguishing NPS that had significant differential diagnostic value in separating MNCD-AD from ScVMNCD as well as MNCD patients from CNEP. The most prevalent NPS in patients with MNCD-AD were anxiety (81.43%) and irritability (67.14%). The most distinguishing NPS in ScVMNCD group were depression (81.43%). In prior three population-based studies (8, 12, 29), which used similar instruments to measure NPS, depression, anxiety, apathy/indifference, irritability/lability, and appetite/eating were the most distinguishing features between the cognitively normal and MNCD (MCI) patients without subdividing them into their etiological types. In the Mayo Clinic Study of Aging, Geda et al. (8) found the prevalence of apathy, agitation, and irritability to be slightly higher in persons with amnestic MCI, while depression, anxiety, delusion, and disinhibition to be slightly higher in persons with non-amnestic MCI. Our findings show that, in MNCD-AD (which clinically is the closest to amnestic single-domain MCI), anxiety and irritability were predominant among NPS, while depression, delusion, and disinhibition were not significant ones. At the same time, depression was more common in ScVMNCD. This etiological MNCD type can be regarded as a multi-domain amnestic MCI. From this point of view, our results overlap with those of Geda et al. (8). In contrast, in the Cache County Study, Peters et al. (12) observed no differences in NPS prevalence between amnestic MCI participants and those with other types of MCI.

We also found significant associations between all meaningful NPS and the differential clinical features in MNCD-AD and ScVMNCD patients. Obtained significant correlation between anxiety/irritability and memory disturbances allowed us to identify amnestic–anxious–irritable syndrome as the main clinical phenotype in patients with MNCD-AD. Similarly, a high correlation of depression and executive dysfunction indicates that dysexecutive–depressive syndrome can be considered as the most prevalent clinical manifestation in patients with ScVMNCD. The differences of cognitive–emotional phenotypes in patients with MNCD-AD and ScVMNCD can be attributed to a variety of underlying neural mechanisms. In MNCD-AD, neurodegenerative changes in some limbic structures (e.g., hippocampus and amygdala) could simultaneously disturb memory and emotional processing leading to decrement in declarative memory, excessive anxiety, and irritability (31, 32). Microvascular alterations of prefrontal-subcortical circuits in ScVMNCD could be crucial for executive impairments (33) and depression (33, 34).

Generally, our data demonstrate that NPS in MNCD are associated with a significantly higher level of functional limitations compared to CNEP and confirm the results of the previous study (10). Moreover, we have shown that patients with ScVMNCD have more severe functional impairment compared to those with MNCD-AD. Our results correspond to those by Hanfelt et al. (6) that cerebrovascular disease leads to more prominent functional disturbances in MCI patients.

A number of potential limitations should be considered when interpreting the results. Our study was limited by a relatively small sample size of patients. Although study power for most of NPS (depression, irritability, sleep disturbances) was approximately 85%, when assessing the prevalence of anxiety between CNEP and ScVMNCD, the power was much lower. Therefore, the results need to be confirmed in population studies. Measurement error for NPS may have occurred (10), even though the NPI has been shown to have good psychometric characteristics (22).

## Conclusion

To our knowledge, this study is the first to investigate the prevalence of the NPS in the main etiological types of MNCD. We found the most distinguishing NPS that had significant differential diagnostic value in separating MNCD-AD from ScVMNCD and MNCD patients from cognitively normal persons. The most prevalent NPS that had significant predictive value for MNCD-AD membership were anxiety and irritability; on contrary, for ScVMNCD membership, that was depression. Obtained significant correlation between distinguishing neuropsychiatric and cognitive symptoms in compared MNCD groups allows us to make a conclusion that amnestic–anxious–irritable syndrome can be the main phenotype in patients with MNCD-AD, as well as dysexecutive–depressive syndrome can be considered as the most prevalent clinical manifestation in patients with ScVMNCD. Early detection of the prominent NPS in MNCD patients may improve the diagnosis, treatment, and course of the disorder. Moreover, the additional consideration of NPS expands MNCD phenotypes and may be used for clinical differentiation of MNCD-AD and ScVMNCD patients. Targeting NPS may also provide important therapeutic avenues for MNCD management.

## Ethics Statement

This study was approved by the local ethics committee and performed in accordance with the ethical standards laid down in the 1964 Declaration of Helsinki and its later amendments. All participants gave written informed consent prior to participation in the study.

## Author Contributions

OL and NC contributed substantially to the conception and design of the study. All authors were involved in the acquisition, analysis, interpretation of the data, and statistical analysis. OL wrote the first version of the manuscript. All the authors contributed to further drafts of the manuscript, critically revised, and approved the final version of the manuscript.

## Conflict of Interest Statement

The authors declare that the research was conducted in the absence of any commercial or financial relationships that could be construed as a potential conflict of interest.

## Abbreviations

AD, Alzheimer’s disease; BADL, the Bristol Activities of Daily Living Scale; CNEP, cognitively normal elderly persons; MCI, mild cognitive impairment; MNCDs, mild neurocognitive disorders; MNCD-AD, mild neurocognitive disorder due to Alzheimer’s disease; NPI, the neuropsychiatric inventory; NPS, neuropsychiatric symptoms; POMA, Tinetti performance-oriented mobility assessment; ScVMNCD, subcortical vascular mild neurocognitive disorder; TIME test, ten-minute intermediate memory evaluation test.
