# Supplementary material for: Neuropsychiatric Symptoms in Patients with the Main Etiological Types of Mild Neurocognitive Disorders: A Hospital-Based Case–Control Study
Source: Front Psychiatry. 2017 May 4;8:75. doi: 10.3389/fpsyt.2017.00075 (PMC5415620; doi:10.3389/fpsyt.2017.00075)
Supplement: Supplementary file 1 [file Table_2.PDF]

Main demographic, cognitive, neurological, and functional characteristics of the comparison groups.

N	Variables	*p* Value		
		**CNEP vs MNCD-AD**	**CNEP vs ScVMNCD**	**MNCD-AD vs ScVMNCD**
1	Age (years)	0.95[a]	0.97[a]	0.99[a]
2	Gender, male/female	0.89[b]	0.21[b]	0.23[b]
3	Education (years)	0.58[a]	0.78[a]	0.94[a]
4	MMSE, score	<0.00001	<0.00001	0.09
5	Delayed recall, TIME test	<0.00001	<0.00001	0.05
6	Delayed cued recall+recognition, TIME test	<0.00001	<0.00001	<0.00001
7	Clock drawing test, part I	0.09	<0.00001	<0.00001
8	Verbal fluency	0.06	<0.00001	<0.00001
9	Kinetic apraxia, Luria's tests	1.0	<0.00001	<0.00001
10	Visuospatial apraxia, Luria's	<0.00001	0.75	0.0003
11	The severity of pseudobulbar syndrome	1.0	<0.00001	<0.00001
12	POMA	0.06	<0.00001	<0.00001
13	BADL	<0.00001	<0.00001	0.00002
**CNEP vs MNCD-AD**				
	↓ labels↓	↓ P-values ↓	P-value's rank (i)	Benjamini-Hochberg P-value<br>$(i/m)*Q$<br>(Q=0.25, m=13)
1	MMSE, score	<0.00001	1	0.019
2	Delayed recall, TIME test	<0.00001	1	0.019
3	Delayed cued recall+recognition, TIME test	<0.00001	1	0.019
4	Visuospatial apraxia, Luria's	<0.00001	1	0.019
5	BADL	<0.00001	1	0.019
6	POMA	0.06	2	0.038
7	Verbal fluency,	0.06	2	0.038
8	Clock drawing test, part I	0.09	3	0.058
9	Education (years)	0.58[a]	4	0.077
10	Gender, male/female	0.89[b]	5	0.096
11	Age (years)	0.95[a]	6	0.115
12	Kinetic apraxia, Luria's tests	1.0	7	0.135
13	The severity of pseudobulbar syndrome	1.0	7	0.135
**CNEP vs ScVMNCD**				
1	MMSE, score	<0.00001	1	0.019

2	Delayed recall, TIME test	<0.00001	1	0.019
3	Delayed cued recall+recognition, TIME test	<0.00001	1	0.019
4	Clock drawing test, part I	<0.00001	1	0.019
5	Verbal fluency	<0.00001	1	0.019
6	Kinetic apraxia, Luria's tests	<0.00001	1	0.019
7	The severity of pseudobulbar syndrome	<0.00001	1	0.019
8	POMA	<0.00001	1	0.019
9	BADL	<0.00001	1	0.019
10	Gender, male/female	0.21[b]	2	0.038
11	Visuospatial apraxia, Luria's	0.75	3	0.058
12	Education (years)	0.78[a]	4	0.077
13	Age (years)	0.97[a]	5	0.096
**MNCD-AD vs ScVMNCD**				
1	Delayed cued recall+recognition, TIME test	<0.00001	1	0.019
2	Clock drawing test, part I	<0.00001	1	0.019
3	Verbal fluency	<0.00001	1	0.019
4	Kinetic apraxia, Luria's tests	<0.00001	1	0.019
5	The severity of pseudobulbar syndrome	<0.00001	1	0.019
6	POMA	<0.00001	1	0.019
7	BADL	0.00002	2	0.038
8	Visuospatial apraxia, Luria's	0.0003	3	0.058
9	Delayed recall, TIME test	0.05	4	0.077
10	MMSE, score	0.09	5	0.096
11	Gender, male/female	0.23[b]	6	0.115
12	Education (years)	0.94[a]	7	0.135
13	Age (years)	0.99[a]	8	0.153

MNCD-AD, patients with mild neurocognitive disorder due to Alzheimer's disease; *ScVMNCD*, patients with mild subcortical vascular neurocognitive disorder; [a] ANOVA, Post-hoc Scheffe test; [b] chi-square test; nonparametric ANOVA test for multiple comparisons if not otherwise specified

Frequency of NPS in CNEP and patients with the main types of MNCD.

N	NPI items	*p* Value		
		**CNEP vs MNCD-AD**	**CNEP vs ScVMNCD**	**MNCD-AD vs ScVMNCD**
1	NPI, total score	<0.00001[a]	<0.00001[a]	0.97[a]
2	Total prevalence of NPS	<0.00001	<0.00001	0.15
3	Agitation	-	-	-
4	Delusion	-	-	-
5	Hallucinations	-	-	-
6	Depression	0.062	<0.00001	<0.0001
7	Anxiety	<0.0001	0.03	<0.0001
8	Euphoria	-	0.37	0.32
9	Apathy	-	<0.00001	<0.00001
10	Disinhibition	-	0.006	0.002
11	Irritability	<0.0001	0.03	<0.0001
12	Aberrant motor behavior	-	0.07	0.04
13	Sleep	<0.0001	0.30	<0.0001
14	Eating/appetite	0.12	0.04	0.47
**CNEP vs MNCD-AD**				
	↓ labels↓	↓ P-values ↓	P-value's rank (i)	Benjamini-Hochberg P-value $(i/m)*Q$ (Q=0.25, m=14)
1	NPI, total score	<0.00001[a]	1	0.017
2	Total prevalence of NPS	<0.00001	1	0.017
3	Anxiety	<0.0001	2	0.036
4	Irritability	<0.0001	2	0.036
5	Sleep	<0.0001	2	0.036
6	Depression	0.062	3	0.054
7	Eating/appetite	0.12	4	0.071
8	Euphoria	-		
9	Apathy	-		
10	Disinhibition	-		
11	Aberrant motor behavior	-		
12	Agitation	-		
13	Delusion	-		
14	Hallucinations	-		

**CNEP vs ScVMNCD**				
1	NPI, total score	<0.00001[a]	1	0.017
2	Total prevalence of NPS	<0.00001	1	0.017
3	Depression	<0.00001	1	0.017
4	Apathy	<0.00001	1	0.017
5	Disinhibition	0.006	2	0.036
6	Anxiety	0.03	3	0.054
7	Irritability	0.03	3	0.054
8	Eating/appetite	0.04	4	0.071
9	Aberrant motor behavior	0.07	5	0.090
10	Sleep	0.30	6	0.107
11	Euphoria	0.37	7	0.125
12	Agitation	-		
13	Delusion	-		
14	Hallucinations	-		
**MNCD-AD vs ScVMNCD**				
1	Apathy	<0.00001	1	0.017
2	Anxiety	<0.0001	2	0.017
3	Depression	<0.0001	2	0.036
4	Irritability	<0.0001	2	0.036
5	Sleep	<0.0001	2	0.036
6	Disinhibition	0.002	3	0.054
7	Aberrant motor behavior	0.04	4	0.071
8	Total prevalence of NPS	0.15	5	0.090
9	Euphoria	0.32	6	0.107
10	Eating/appetite	0.47	7	0.125
11	NPI, total score	0.97[a]	8	0.143
12	Agitation	-		
13	Delusion	-		
14	Hallucinations	-		

CNEP, cognitively normal elderly persons*; MNCD-AD*, patients with mild neurocognitive disorder due to Alzheimer's disease*; ScVMNCD*, patients with mild subcortical vascular neurocognitive disorder; [a] nonparametric ANOVA test for multiple comparisons; chi-square test if not otherwise specified
